# Harnessing Mammalian- and Plant-Derived Exosomes for Drug Delivery: A Comparative Review

**DOI:** 10.3390/ijms26104857

**Published:** 2025-05-19

**Authors:** Shynggys Sergazy, Sergazy Adekenov, Ilya Khabarov, Kymbat Adekenova, Assiya Maikenova, Mohamad Aljofan

**Affiliations:** 1 Research and Production Center Phytochemistry, Gazaliyev Street, Karaganda 100009, Kazakhstan; shynggys.sergazy@gmail.com (S.S.); arglabin@phyto.kz (S.A.); i.khabarov@phyto.kz (I.K.); kymbat.a@inbox.ru (K.A.); maikenova98@gmail.com (A.M.); 2 National Laboratory Astana, Center for Life Sciences, Nazarbayev University, Astana 010000, Kazakhstan; 3 Department of Biomedical Sciences, Nazarbayev University School of Medicine, Astana 010000, Kazakhstan

**Keywords:** exosomes, drug delivery, plant-based exosomes, mammalian-derived extracellular vesicles

## Abstract

Exosomes, nanoscale vesicles involved in intercellular communication, have garnered significant attention for their potential in drug delivery and therapeutic applications. This review provides a comparative analysis of mammalian-derived exosomes, particularly milk-derived exosomes, and plant-derived exosome-like nanoparticles (PDENs). It explores their biogenesis, bioactivities, and functional similarities, including their roles in cellular communication, immune modulation, and disease therapy. While milk-derived exosomes exhibit promising biocompatibility and stability for targeted delivery, PDENs offer distinct advantages, such as scalability and inherent bioactivities, derived from their plant sources. Despite similarities in their structure and cargo, PDENs differ in lipid composition and protein profiles, reflecting plant-specific functions. Emerging research highlights the therapeutic potential of PDENs in managing inflammation, oxidative stress, and other diseases, emphasizing their utility as functional food components and nanocarriers. However, challenges related to their chemical stability and large-scale production require further investigation. This review underscores the need for advanced studies to fully harness the potential of these natural nanocarriers in drug-delivery systems and therapeutic interventions.

## 1. Introduction

Exosomes are small vesicles with a diameter of 40–150 nm (on average, about 100 nm), produced by multivesicular bodies of most animal and human cells into the extracellular region [1,2,3,4]. They are encased in a lipid bilayer and membrane proteins, and contain various cytosolic components such as proteins and RNA, miRNA, and DNA [5]. The biogenetic, architecture, components, and molecular processing of exosomes mirror that of their cells of origin; hence, they might provide valuable information about the parent cell [6,7]. Having the same topology as in the parent cells, exosomes can carry chemical cargo with harmless characteristics into the biological environment and perform different functions, such as transmitting signals to recipient cells and recognizing antigen-presenting molecules during intercellular communication [8].

They are considered to be a major component of the cellular communication signaling pathway and are being explored as a potential means of conducting targeted drug delivery. The naturally occurring nanoparticles are able to penetrate deeply into tissues and overcome barriers such as the blood–brain barrier, placenta, and cytoskeleton [8]. Notably, they possess a slightly negative zeta potential, ensuring their prolonged circulation and optimal distribution [9]. Furthermore, some exosomes can evade the immune system, exhibiting low immunogenicity and high stability in the bloodstream, thus prolonging the circulation of drugs within the body [10].

Therefore, exosomes may serve as nanocarriers to deliver various small bioactive molecules. For instance, bovine-milk-derived exosomes, protected from degradation by stomach acids, are considered promising candidates for targeted drug delivery [1,11]. Milk-derived exosome-encapsulated formulations hold potential for therapeutic purposes and viewed as suitable new drug carriers for reaching distant tissues [12,13,14]. Among milk-derived exosomes, those from cow’s milk have been most extensively characterized [15]. It is now evident that milk can become a unique source of exosomes, paving the way for drug transport systems derived from milk exosomes [13]. For example, the pharmaceutical company Roche agreed to pay PureTech Health up to 36 million US dollars for access to exosomes extracted from dairy cow milk [16]. The possibility of using milk-derived exosomes for delivering biologically active molecules makes the study of milk-derived exosomes highly relevant. However, there are concerns that mammalian milk may not be an ideal source of exosomes for medical purposes due to the potential for disease transmission.

Recently, plant-based exosomes have been investigated as a potentially rich source of usable exosomes as well. Plant cells were reported to secrete exosome-like vesicles, which are likely used as a method of intercellular communication and immune regulation to protect plants from pathogen invasions [9]. Plant-derived exosome-like nanoparticles (PDEN) are similar to exosomes of animal origin in terms of properties such as size distribution, surface electrical charge, morphology, density, and some components [17]. In this review, we attempt to compare and contrast mammalian- and plant-derived exosomes and their potential uses as drug-delivery systems.

## 2. Milk-Derived Exosomes

### 2.1. Biogenesis

**Figure 1:**
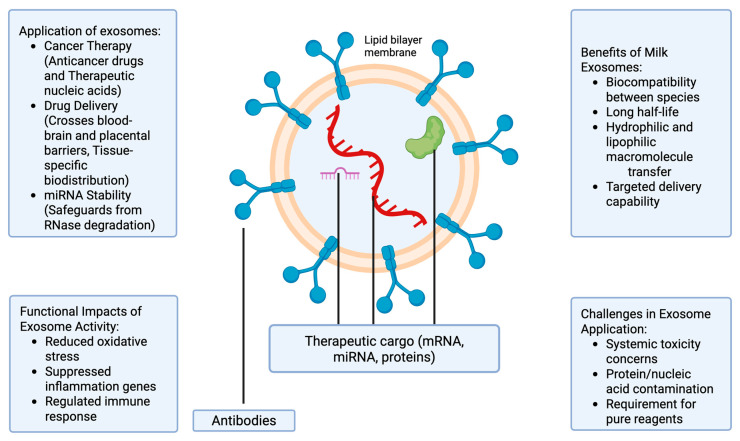
Schematic representation of milk-derived exosomes. Milk-derived exosomes are lipid bilayer-bound nanovesicles secreted into milk, containing bioactive cargos such as proteins (tetraspanins, heat shock proteins), lipids (cholesterol, sphingomyelin, phosphatidylserine), and nucleic acids (miRNAs, mRNAs). Their structure facilitates biostability and interspecies compatibility, protecting encapsulated molecules from enzymatic degradation. Functionally, they participate in cellular communication, immune modulation, and serve as efficient vehicles for delivering therapeutic agents like chemotherapeutics, siRNAs, and antioxidants across biological barriers (e.g., blood-brain barrier). Their high biocompatibility and bioactivity make them promising platforms for drug delivery and regenerative medicine.

The process of milk-derived exosome release is quite complex and tightly regulated by different signaling pathways [18]. They are thought to be primarily released by endocytosis, forming multivesicular bodies (MVBs) which fuse with the plasma membrane, releasing exosomes into the extracellular space, and sort specific proteins, lipids, and RNA into intraluminal vesicles (ILVs) within MVBs [18]. Generally, milk-derived exosomes contain proteins, nucleic acids, and lipids [19]. The latter includes sphingolipids, cholesterol, phosphatidylserine, and ceramide, which contribute to their membrane structure and stability. The reported proteins that were found in milk-derived exosomes including tetraspanins (CD9, CD63, CD81), heat shock proteins (HSP70, HSP90), and other membrane-associated proteins, as well as enzymes like proteases and kinases [19]. These proteins often serve functional roles in intercellular communication and cargo recognition [19]. Another important component of milk-derived exosome is nucleic acid, including microRNAs (miRNAs), mRNAs, and long non-coding RNAs (lncRNAs), which are involved in gene regulation and cellular communication [19]. Figure 1 describes the structure and potential uses of milk-derived exosomes.

#### Key Regulatory Factors in Mammalian Exosome Formation and Release

The formation of mammalian-derived exosomes is primarily governed by the Endosomal Sorting Complex Required for Transport (ESCRT)-dependent and ESCRT-independent pathways. The ESCRT machinery, consisting of ESCRT-0, -I, -II, and -III complexes, orchestrates membrane invagination and cargo sorting into intraluminal vesicles (ILVs) within multivesicular bodies (MVBs) [20]. Additionally, tetraspanins (CD63, CD81) and ceramide-mediated pathways play essential roles in ESCRT-independent exosome biogenesis [21]. Release is often modulated by Rab GTPases (Rab27a/b, Rab11) which regulate MVB trafficking and fusion with the plasma membrane [22].

### 2.2. Therapeutic Use of Milk-Derived Exosomes

Milk exosomes show great potential in the development of new therapeutic agents for the treatment of a range of disorders, including cancer [23]. Nonetheless, the current literature lacks conclusive data regarding the use of milk-derived vesicles, especially exosomes, to deliver bioactive molecules to cells [24]. For the application of milk exosomes in cancer therapy, two paths can be distinguished: the use of anticancer drugs and the use of therapeutic nucleic acids [13,25,26]. The side effects of cytotoxic and cytostatic agents are some of the prevailing concerns in cancer chemotherapy [23]. In fact, cells with a significant mitotic index are more susceptible to cytostatic action; therefore, the application of cytostatic agents causes the death of tumor cells and can interfere with the skin, bone marrow, hair, and epithelium cells growth [27]. However, targeted chemotherapeutic agents may be an effective solution for the problem of toxic effects during chemotherapy [28]. Since exosomes have mRNA, microRNA, and proteins that can damage cells, using exosomes as delivery agents remains controversial. Various studies focusing on protein and nucleic elements of exosomes have overestimated the quantity of proteins or nucleic acid molecules in exosomes because of drug contamination with isolating molecules [29]. For this reason, it is necessary to use pure reagents to determine biopolymers, which are internal components of exosomes. Potentially, exosomes can be utilized as encapsulation for drugs, where therapeutic nucleic acid is applied on the surface or after “loading” the constituents through sonication or electroporation [30]. Alternatively, a combination of exosomes with liposomes is a viable approach, but only if the necessary contents of “chimeric” exosomes are used [31].

Nevertheless, exosomes’ characteristics such as their biocompatibility between species, a longer half-life cycle, self-incorporation by other cells, and the possibility of macromolecules’ transfer from both hydrophilic and lipophilic sources make them a promising drug-delivery system [32]. Further, exosomes can cross biological barriers, including the blood–brain and placental barriers, which allows the delivery of therapeutic molecules to otherwise inaccessible tissues [33,34,35]. Their surfaces can also be designed for targeted delivery, hence facilitating tissue-specific biodistribution [36]. Table 1 provides an overview of some therapeutic applications of exosomes, along with their mechanisms of action.

Among the various cargoes transported in milk exosomes, there are many non-coding RNAs, in particular microRNAs (miRNAs) [1]. An interesting factor is that exosomes appear to increase miRNAs’ stability by safeguarding them from RNase degradation [32]. MiRNAs are small RNAs, measuring 19 to 22 nucleotides in length, which can control gene expression at the post-transcriptional stage [1]. However, the question of whether or not miRNAs that are naturally delivered in exosomes can apply genome regulation remains elusive. To overcome this, it might be necessary to include a minimum number of miRNA samples for individual cells.

The application of anticancer drugs on the surface of natural, biologically active components, including proteins, considerably enhances the therapy’s suitability [41]. In recent times, significant focus has been directed towards the capacity of exosomes to address physiological limitations, for example the blood–brain constraint [23]. Various experts have even tried to utilize exosomes isolated from distinct cell lines for selected drug delivery [8]. Despite major results, the development of exosomes as therapeutic agents presents key challenges. Studies have shown that, although exosomes can be derived from cow’s milk in large quantities, concomitant administration of milk exosomes failed to induce systemic toxicity or anaphylactic impacts in rat models [23]. In addition, exosomes from non-loaded camel milk reduced oxidative stress while suppressing several genes involved in inflammation and the induction of immune responses [42].

#### Mechanisms Underlying Superior Targeting by Milk-Derived Exosomes

Milk-derived exosomes interact with target cells through multiple mechanisms, including ligand–receptor binding, membrane fusion, and endocytosis. They express abundant adhesion molecules, such as integrins (α6β4, α6β1) and tetraspanins (CD63, CD81), facilitating specific binding to cellular receptors like ICAM-1 and enhancing internalization [43]. Moreover, the presence of phosphatidylserine on their surface aids in recognition through phagocytic cells [44]. Compared to PDENs, milk-derived exosomes also possess higher membrane fluidity and optimized lipid raft domains that facilitate rapid membrane fusion and cytoplasmic cargo release [45]. These properties confer superior targeting and uptake efficiency compared to plant-derived vesicles.

## 3. Promising Developments in Biomedical Applications of Milk-Derived Exosomes

### 3.1. Impact on Nervous System

**Figure 2:**
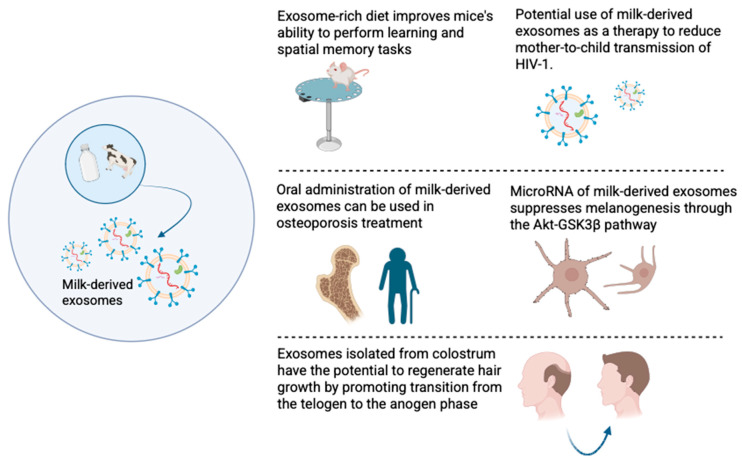
Promising potential biomedical applications of milk-derived exosomes.

Milk-derived exosomes have shown great promise in biomedical applications (Figure 2). The study performed by Zhou et al. found that small extracellular vesicles (sEVs) isolated from milk can accumulate in different parts of the brain, including the hippocampus, cortex, and cerebellum. In vitro experiments on bEnd.3 mouse brain endothelial cells internalize sEVs via a saturable transport mechanism and secrete microRNA cargoes across the basal membrane [46]. A subsequent study showed that the exosome-rich diet (ERS) greatly changed hippocampal gene expression; 295 genes were differentially expressed when compared to mice on an exosome-depleted diet (ERD). Morphological studies revealed that animals consuming a low quantity of exosomes had reduced dendritic complexity of dentate granule cells in the hippocampus [46]. This group had ninefold lower results in the Barnes maze test, which measures spatial learning and memory. Additionally, mice that received the ERS diet showed higher resistance to seizures from kainic acid administration. Based on the results obtained, the authors highlighted the need for dairy-derived exosomes in infant nutrition and explained how they influence subsequent development [46].

### 3.2. Antiviral Potential of Milk-Derived Exosomes

The ability of extracellular vesicles of milk origin to protect the body from the influence of various viruses is extremely poorly studied. However, the fact that these exosomes have immunomodulatory properties on T cells has led to the theory that milk-derived EVs and exosomes can play a role in building the immune system of newborns [47]. Näslund et al. have found that EVs obtained from breast milk can bind to DC-SIGN receptors, which subsequently prevents binding of HIV-1 to dendritic cells that are produced from monocytes and limits the spread of the virus to CD4 T cells [48]. Additionally, goat-milk-derived exosomes were observed to have antiviral activity against the dengue virus and Newcastle disease virus strain Komarov (NDV-K) [49]. The ability to resist and protect from the dengue virus lies in the inhibition of NS3 expression, replication of the viral genome, and maturation of viral molecules. Goat-milk-derived exosomes inhibited NDV-K as well, but did not show any significant effect against HIV-1. Yenuganti et al. suggested that HIV-1 was not inhibited because antiviral activity might be virus-specific [49].

### 3.3. Milk-Derived Exosomes Promote Hair Growth

It has been found that exosomes isolated from bovine colostrum have significant potential in enhancing hair growth. Exosomes increased the proliferation of human hair dermal papillary (DP) cells [50]. These cells control hair growth, shape, size, and color, and send signaling molecules to epithelial cells after the maturation of the hair follicle [51]. The increase in DP cell proliferation and acceleration of hair regeneration by exosomes occur due to the activation of the Wnt/β–catenin pathway. It was also noted that lactoferrin was highly expressed in milk exosomes, which may have an additional positive effect on stimulating hair growth. A comparison of the effectiveness of exosomes with the effectiveness of minoxidil was also conducted. The results indicate that exosomes have comparable positive results, but do not have the side effects of minoxidil [50].

### 3.4. Milk-Derived Exosomes and Bone Health

Osteoporosis is a common disease among the aging population and is considered to be the most common metabolic bone disease in the world [52]. For this reason, there is an active search for treatment methods, and interest in milk-derived exosomes is actively growing. For instance, in a study by Hao et al. on a mouse model of ovariectomy-induced osteoporosis, bovine-milk-derived exosomes had a protective effect on bone tissue [53]. Another similar study showed that exosomes improved the stiffness and microstructure of the femur and reduced the number of osteoclasts [54]. A decrease in the RANKL/OPG ratio in bone tissue and serum was also noted [54]. There is also a relationship between improved gut microbiota health and bone health [55]. The administration of exosomes helped to strengthen the barrier function of the stomach by increasing the growth of Bacteroides bacteria, which are responsible for the production of short-chain fatty acids, including acetic and propionic acids. These short-chain acids themselves play an important role in maintaining bone health [55].

### 3.5. Cosmetics Applications of Milk-Derived Exosomes

In addition to the use of milk exosomes in the treatment of various diseases, these particles can also be actively used in the beauty industry to create new, modern cosmetic products. For instance, milk-derived exosomes have been shown to successfully inhibit TYR and reduce melanin content in melanoma cells and melanocytes [56]. These effects were mediated by miR-2478 from milk exosomes, which functions as a regulator of melanogenesis by directly targeting Rap1a. These properties allow milk-derived exosomes to be used in the manufacture of whitening skincare products [56]. Another example is the use of exosomes in the fight against signs of aging. The main cause of aging is a decrease in collagen. This is mainly due to changes in the dermal extracellular matrix [57]. Lu et al. studied the effect of exosomes isolated from milk on keratinocytes and fibroblasts and found that exosomes increased the expression of FLG, CD44, and HAS2 genes, which are responsible for skin hydration, and also observed a reduction in skin wrinkles [57].

## 4. Plant-Derived Exosome-like Nanoparticles

### 4.1. Biogenesis

**Figure 3:**
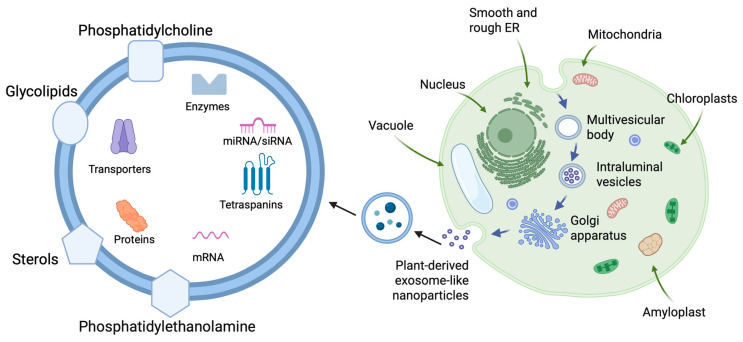
Schematic representation of plant-derived exosome-like nanoparticles (PDENs) and their formation pathway. Formation of PDENs may go through the multivesicular bodies (MVBs) pathway. The process involves inward budding of plasma membrane, resulting in the formation of early endosomes that mature and interact with the trans-Golgi network apparatus. Consequently, this leads to the formation of MVBs. MVBs fuse with the cytoplasmic membrane and release intraluminal vesicles (ILVs) into the outer environment. Released ILVs are classified as PDENs [73].

Plant-derived exosomes are believed to originate from pathways similar to those of mammalian exosomes (Figure 3) [58]. While the exact mechanisms of exosome release in plants are not fully known, evidence suggests that exosomes might be influenced by environmental stressors and/or pathogen recognition [59]. Generally, the process is thought to involve endocytosis and the formation of MVBs, although the specific mechanisms in plants are less well characterized than in mammals [60]. Interestingly, the formation process of plant-derived exosomes appears to involve the trafficking of small vesicles through the Golgi and trans-Golgi network, where they may also bud from the endosomal compartments [61]. However, plant cells do not possess the specific endosomal sorting complexes required for transport (ESCRT) machinery commonly seen in mammal cells; however, the process might also involve alternative formation [62]. Like mammalian-derived exosomes, plant-derived exosomes also contain lipids, proteins, and nucleic acids [63]. A summary of some of the reported differences between milk-derived exosomes and PDENs is provided in Table 2. Plant exosomes contain typical plant lipids, including phospholipids (phosphatidylcholine, phosphatidylethanolamine), glycolipids, and sterols [61]. While they are similar in composition to mammalian-derived exosomes, plant-derived exosomes have unique lipid profiles that are reflective of the cellular membranes of plants [61]. Interestingly, they are thought to contain a wide range of proteins, including enzymes, transporters, and proteins such as tetraspanins and other plant-specific proteins, which are reported to be involved in stress responses [62]. Similarly, plant-derived exosomes carry a range of RNAs, including small RNAs (miRNAs, siRNAs), mRNA, and potentially other non-coding RNAs involved in gene regulation and signaling [59].

Thus, different plants can be used to obtain exosomes, including berries, fruits, vegetables, etc. Depending on the source material, plant-based exosomes can differ in their properties, as they contain unique cargo inherited from the original plant [64]. For instance, preclinical studies have shown that grape exosomes have high anti-inflammatory activity, while exosomes isolated from strawberries have shown an ability to reduce oxidative stress [65,66]. Isolation methods for producing PDENs do not differ significantly from techniques that are applied to isolate exosomes from milk or other mammalian cells. Generally, plant tissue is homogenized before exosome isolation, and later obtained biomass can undergo ultracentrifugation, density gradient centrifugation, size-exclusion chromatography, or polymer-based precipitation, depending on the chosen protocol for isolation [67].

**Table 2:** Differences between PDENs and milk-derived exosomes.

Function	Milk-Derived	References	PDENs	References
Cellular communication	They mediate cell–cell communication via transferring molecular signals such proteins, lipids, and RNAs.	[68]	They facilitate cellular signaling by transferring lipids, proteins, and RNAs between different cells during stress responses or developmental processes.	[60]
Biological response regulation	They are involved in immune response including antigen presentation and the modulation of immune cell activity.	[69,70]	They are reported to regulate cellular responses to drought, salt stress, and immunity.	[63]
Disease modulation	They play a role in cancer progression. Tumor-derived exosomes might facilitate metastasis.	[71,72]	They regulate plant pathogen immune responses by transferring immune-related molecules like plant-specific small RNAs.	[59]

#### Key Regulatory Factors in Plant-Derived Nanoparticle Formation

In plants, the regulatory mechanisms for exosome-like vesicle formation are less characterized compared to those in mammals. While ESCRT components are partially conserved, alternative pathways are likely to exist. Key regulators include TET8 (a tetraspanin-like protein in Arabidopsis) associated with exosomal secretion under pathogen stress [59]. SNARE proteins and Rab GTPases (e.g., Rab11 homologs) may also influence MVB formation and vesicle trafficking [62]. Stress-related signaling (e.g., salicylic acid pathways) appears to enhance vesicle release, suggesting environmental stimuli as a key modulator [60].

### 4.2. Therapeutic Use of PDENs

PDENs have demonstrated a range of therapeutic properties, including antioxidant [17], anti-inflammatory [37], anticancer [38], and antibiotic effects [74,75]. By analogy with milk exosomes, PDENs could hypothetically be used either as transport vesicles or as independent therapeutic substances. Interestingly, the structural and functional biomolecules they contain could also be useful in clinical applications [39]. To date, PDENs have been obtained from a variety of edible plants, including grapes, grapefruit, ginger, lemon, broccoli, carrots, coconuts, and apples [76]. Importantly, it is safe to assume that they may have beneficial therapeutic effects due to natural biochemicals in the source plants. PDENs derived from grapes, blueberries, and strawberries exhibited inherent biological activity (antioxidant, cytoprotective, anti-inflammatory, anti-proliferative) in vitro and, moreover, have the ability to deliver various molecules in a targeted fashion [39]. There is no evidence of inflammation or toxicity associated with the use of PDENs, and they are considered to be relatively safe [63]. Studies have demonstrated that PDENs derived from fruits like grapes and blueberries exhibit antioxidant and anti-inflammatory activities in vitro, highlighting their potential as therapeutic nanocarriers.

Although research on PDENs has only recently begun, there are several earlier researchers who have described the biogenesis and mechanisms of PDENs. Early evidence suggests that plants produce PDENs in response to multiple biotic and abiotic environmental stresses, including infection [76]. PDENs have been shown to be taken up by intestinal macrophages and are capable of exhibiting cross-species communication by inducing multiple cytokines [76]. Some studies have shown that PDENs may provide health benefits, so it would be interesting to apply them as a new functional food ingredient. However, the stability of PDEN in food digestion, processing, and storage must be clearly demonstrated before they can be used in functional foods [76].

Studies have recently shown that, when taken orally, PDENs penetrate into the colon [76] and, depending on the source of the PDENs, remain in the colon area, facilitating intestinal function, providing an anti-inflammatory effect, or penetrating into liver cells, exerting a hepatoprotective effect there [39]. These results demonstrate the effectiveness of PDENs as ingredients in functional foods to alleviate various diseases. PDENs are currently being investigated for their suitability as an alternative to milk exosomes as drug carriers. In terms of large-scale production capabilities, PDENs have great potential for applications in disease therapy as well as for the development of drug nanocarriers that are capable of delivering different doses depending on their physiological, chemical, and biological characteristics. The yield of PDENs that can be extracted from plants is much higher than that of exosomes from mammalian cell cultures, indicating their cost-effectiveness as nanofactories [39]. For example, Wang et al. first developed PDENs as nanovectors for delivering therapeutic agents to brain tumors [77]. They showed that PDENs accumulate in certain tissues in vivo and circulate for a long time in the peripheral blood due to the high stability of the nanovectors.

Plant biomolecules have attracted much attention due to their ability to improve health and provide protection against various diseases. PDENs are naturally generated and carry harmless components from parent cells, some of which have been shown to possess therapeutic effects. In addition, PDENs can be intrinsically localized to target tissues, one of the most important features of a targeted delivery system. PDENs exhibit intrinsic targeting capabilities owing to their specific lipid and protein composition, including surface molecules that facilitate selective uptake by intestinal epithelial cells and macrophages [78]. However, PDEN is a new concept in nanomedicine, and not all its aspects have been fully identified and described. There remains a need to expand the use of innovative approaches to drug development to more effectively and efficiently treat various diseases.

Research on PDENs is still mostly carried out at the in vitro level, which opens up more opportunities for comprehensive research, especially regarding their biological activity, isolation, handling, and standardized mass production. Previous studies have assessed, under preclinical conditions, the effects of polyphenol concentrates from cranberries, lingonberries, bilberries, blueberries, and grapes in vitro and in vivo on several models of age-associated pathology [79]. According to research data, polyphenolic extracts of grapes and blueberries had the most pronounced therapeutic potential [80,81]. This circumstance initiated further research into the possibility of increasing the biological activity of polyphenols through the use of recently discovered exosome-like nanoparticles of plant origin.

#### Disease Models and Clinical Translation Potential of PDENs

Preclinical studies have demonstrated the therapeutic potential of PDENs in several disease models, notably in inflammatory bowel disease (using ginger-derived nanoparticles) [37], liver fibrosis (using grapefruit-derived nanoparticles) [74], and colon cancer (using lemon- and grape-derived nanoparticles) [39]. The next translational steps include establishing standardized extraction and purification protocols, ensuring batch-to-batch reproducibility, evaluating long-term biosafety, and conducting rigorous pharmacokinetic and biodistribution studies. Additionally, controlled clinical trials are essential in validating the efficacy and safety of PDEN-based therapies in humans.

## 5. Promising Developments in Biomedical Applications of PDENs

### 5.1. Anticancer Effect of Plant-Derived Exosomes

**Figure 4:**
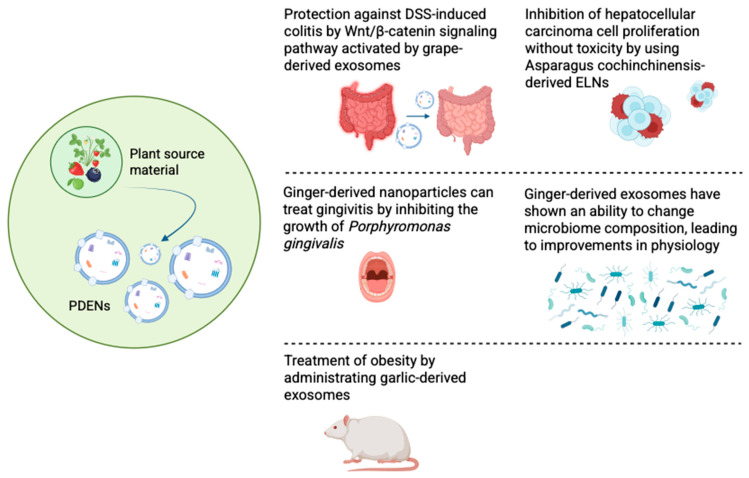
Promising potential biomedical applications of PDENs.

Numerous studies have shown PDENs’ potential to fight a wide range of diseases (Figure 4). One of the most interesting and exciting areas is the anticancer properties of exosomes. For instance, PDENs isolated from Asparagus cochinchinensis were found to inhibit hepatocellular carcinoma cell proliferation, induce cellular apoptosis, and upregulate factors related to apoptosis in both in vitro and in vivo studies without causing any adverse side effects [82]. Exosomes derived from the juice of fresh tea leaves were co-incubated for 5 h with breast tumor cells; afterwards, it was discovered that more than 80% of the cancer cells absorbed the nanoparticles [83]. An increased amount of reactive oxygen species induced by absorbed exosomes has led to the damage of mitochondria, cell cycle arrest, and, finally, the apoptosis of cancer cells [83]. Additionally, the next in vivo experiments have demonstrated no toxicity in the oral administration of tea leaf nanoparticles. Nanoparticles were successfully absorbed by the small intestine to modulate microbiota and regulate tumor gene expression profiles, achieving the desired therapeutic effect in the fight against breast cancer [83]. It was possible to achieve outstanding results in the fight against gastric cancer using exosomes isolated from lemons [84]. Their anticancer activity is associated with the ability to promote an increase in ROS, which upregulates GADD45a gene expression, ultimately leading to S-phase arrest and apoptosis. The biosafety of lemon-derived exosomes was also evaluated. A comparison of the organs for the morphological normality of the histological sections of the main organs was made. The obtained results suggest that the use of these nanoparticles does not harm the body [84].

### 5.2. Treatment of Periodontitis

Periodontitis is a chronic, multifactorial inflammatory condition linked to the buildup of dental plaque. It is marked by the progressive destruction of the structures that support the teeth, such as the periodontal ligament and alveolar bone [85]. The Gram-negative anaerobic bacteria Porphyromonas gingivalis is the main pathogen that influences the risk of periodontitis infection and its progression. It is capable of creating comfortable conditions for the colonization of oral surfaces, the degradation of periodontal tissues, the induction of destructive immune responses, and growth in a peptide- and hemin-rich inflammatory microenvironment [86]. A specific ratio of phosphatidic acid in ginger-derived nanoparticles has been found to interact with proteins of Porphyromonas gingivalis, namely, bind to HBP35 protein on the surface of bacteria, leading to the inhibition of bacterial growth and a decrease in the incidence of gingivitis [86].

### 5.3. Alteration in Microbiome Composition

PDENs have great significance in digestive system diseases. They are highly stable and can persist in the digestive system, exhibiting their therapeutic properties and successfully resisting digestion by various enzymes [73]. The results of the study conducted by Teng et al. revealed that exosome-like nanoparticles isolated from ginger were mainly taken up by Lactobacillaceae in a lipid-dependent GELN-mediated manner and contained microRNAs that target various genes of Lactobacillus rhamnosus [87]. One such microRNA, mdo-miR7267-3p, suppresses the ycnE gene, causing the bacteria to induce production of indole-3-carboxaldehyde (I3A). This substance can activate a special receptor (AHR), which leads to the production of the protein IL-22, consequently resulting in strengthening the protective barrier of the intestine. Additionally, the co-incubation of ginger exosome-like nanoparticles with bacteria from the Lactobacillus family showed that they promoted the growth of beneficial strains L. reuteri and L. murinus [87].

### 5.4. Treatment of Obesity

Treating obesity caused by chronic inflammation is a major challenge. One of the features of this disease is the accompanying inflammation of the brain, which is transmitted through the gut–brain axis [88]. The ability of exosomes to penetrate the BBB and circulate in both directions makes them promising in the treatment of inflammation. Thus, it was discovered that garlic-derived exosomes reversed HFD-induced brain inflammation and obesity in a mouse model of HFD-induced obesity via oral administration [89] The brain inflammation process was suppressed through the IDO1-mediated AHR pathway and the c-Myc-regulated c-GAS/STING inflammatory pathway when garlic-derived exosomes were taken up by microglial cells [89]. Pang et al. also found that kidney-bean-derived exosome-like nanovesicles were able to alter gut microbiota and thereby improve symptoms of high-fat-diet-induced obesity [90]. The effect of the nanoparticles was reflected in the growth of beneficial bacteria such as Lactobacillus, Romboutsia, Bifidobacterium, and Ruminococcus. A significant decrease in the excess of Proteobacteria and Desulfobacterota, bacteria whose high presence is often associated with intestinal diseases, was also noted [90,91,92]. Pang et al. suggested that legumes may have antihyperlipidemic, antiobesity, and antidiabetic activity due to the selective enrichment of bacteria [90].

### 5.5. Treatment of Colitis

The therapeutic potential of grape-derived exosome-like nanoparticles has been studied in the treatment of colitis [65]. It was found that the oral administration of grape exosomes to mice resulted in a significant reduction in colonic inflammation induced by dextran sodium sulfate (DSS). The decrease was due to the induction of Lgr5^hi^ intestinal stem cells. Treatment with grape-derived exosomes activates the Wnt/β–catenin pathway and induces the expression of genes that regulate the growth of stem cells. It is assumed that grape nanoparticles are absorbed through the intestinal wall and deliver bioactive molecules that promote stem cell proliferation and tissue regeneration [65].

## 6. Use of Extracellular Vesicles as Drug-Delivery Systems in Diseases

Extracellular vesicles (EVs), including exosomes, have gained significant attention as potential drug-delivery systems due to their intrinsic ability to transfer bioactive molecules between cells. These vesicles are naturally secreted by various cell types and serve as carriers for proteins, lipids, and nucleic acids, making them attractive candidates for therapeutic applications [20]. Their biocompatibility, ability to traverse biological barriers, and potential for targeted delivery have positioned them as promising alternatives to conventional drug carriers [93].

One of the most compelling advantages of EVs is their ability to deliver therapeutic agents with high specificity while minimizing off-target effects. Unlike synthetic nanoparticles or liposomes, EVs exhibit low immunogenicity and enhanced cellular uptake due to their endogenous nature [94]. Additionally, their capacity to cross the blood–brain barrier makes them ideal candidates for treating neurological disorders such as Alzheimer’s disease and glioblastoma [95]. Recent studies have explored the use of mesenchymal-stem-cell-derived EVs (MSC-EVs) in regenerative medicine, demonstrating their potential in modulating inflammatory responses and promoting tissue repair [96]. The delivery of small molecules, such as curcumin and paclitaxel, using EV-based systems has also shown enhanced bioavailability and therapeutic efficacy in preclinical models of inflammatory and cancerous diseases [8].

Therapeutic applications of EV-mediated drug delivery span multiple disease domains, including oncology, neurology, cardiology, and inflammatory disorders. In cancer therapy, exosomes have been employed to deliver chemotherapeutic drugs directly to tumor cells, improving drug retention and minimizing systemic toxicity [97]. Phase I clinical trials investigating dendritic-cell-derived exosomes pulsed with tumor antigens have demonstrated their ability to elicit anti-tumor immune responses in patients with non-small-cell lung cancer. In neurodegenerative diseases, EVs are being explored as delivery vectors for neuroprotective agents, with preclinical studies indicating their potential to modulate neuroinflammation and promote neuronal survival [97]. Additionally, stem-cell-derived EVs have been investigated for their role in cardiac regeneration following myocardial infarction, showing promising results in preclinical settings [98,99].

Several clinical trials have been initiated to evaluate the safety and efficacy of EV-based therapies. A notable Phase I trial assessed the use of MSC-derived exosomes for acute ischemic stroke, focusing on their neuroprotective effects and capacity to reduce inflammation [97]. Another trial investigated plant-derived exosome-like nanoparticles for the targeted delivery of therapeutic RNAs in colon cancer treatment [8]. Furthermore, exosome-based curcumin delivery has been explored for its anti-inflammatory properties in the treatment of inflammatory bowel disease [96]. Despite these promising developments, challenges such as the standardization of EV isolation techniques, the scalability of production, and regulatory hurdles remain significant obstacles to their widespread clinical implementation [20]. Establishing robust protocols for EV characterization and functional assessment is crucial in ensuring consistency and reproducibility in therapeutic applications. The growing body of research on EVs as drug delivery vehicles underscores their transformative potential in modern medicine. Table 3 provides an overview of ongoing clinical trials investigating the use of exosomes in disease treatment, highlighting their therapeutic mechanisms and target conditions.

## 7. Discussion

Extracellular vehicles including exosomes, microvesicles, and apoptotic vesicles, are membrane-bound small structures that are released from cells into the surrounding environment [2]. They play essential roles in intercellular communication, particularly exosomes, which contain several constituents from the cells that secrete them, and appear to be involved in the pathogenesis of various disorders, including cancer, neurodegeneration, and inflammatory diseases [1]. Interestingly, the biocompatibility of exosomes, their circulating stability, and their bioavailability in vivo allowed them to gain increasing attention as an emerging drug-delivery methodology as well as translatable therapeutics over the last decade [3]. Exosomes of animal origin, particularly those derived from milk, have been explored as targeted drug-delivery systems. Recently, our research group at the National Laboratory, Astana-Nazarbayev University, is the first to extract and characterize mare-milk-derived exosomes [40]. We showed that exosomes could improve drug affinity and bioavailability [40]. PDENs, a relatively new area of research, are nanostructures originating from plants that mimic the characteristics of exosomes. Evidence is emerging for the intrinsic biological activity of PDENs, leading to discussions on potential advantages in the development of transport systems. For example, polyphenols, naturally occurring compounds, face challenges related to bioavailability, making the incorporation into PDENs an attractive strategy. While reports indicated that chemical stability and tissue absorption of PDENs appear to be less optimal compared to its milk-derived counterpart [105], we showed that PDENs obtained from polyphenol-rich berries such as grapes, blueberries, and strawberries may enhance antioxidant/radical scavenging activity and exert cytoprotective effects [106]. Thus, we can safely speculate that PDENs could serve as a reliable drug-delivery method to enhance drug bioavailability.

In recent years, PDENs have gained attention as substances possessing intrinsic biological activity and/or serving as vesicles for targeted drug delivery. The description of the primary physicochemical parameters and in vitro biological characteristics of PDEN isolated from polyphenol-rich berries will provide evidence for the potential of PDEN as novel bioactive substances of plant origin and nanocarriers for targeted drug delivery, particularly for enhancing the bioavailability of biomolecules that otherwise would have low bioavailability.

## 8. Future Directions

To fully realize the therapeutic potential of exosomes and PDENs, several key areas require further exploration. First, detailed mechanistic studies are necessary to elucidate the precise pathways governing vesicle biogenesis, cargo selection, and tissue-specific targeting. Second, advances in scalable, Good Manufacturing Practice (GMP)-compliant production methods are critical for clinical translation. Third, bioengineering approaches, including surface modification and cargo loading optimization, should be employed to enhance targeting efficiency and therapeutic payload delivery. Fourth, comprehensive in vivo pharmacokinetic, immunogenicity, and toxicity profiles must be established for both mammalian- and plant-derived vesicles. Future clinical trials should focus on chronic inflammatory diseases, neurodegenerative conditions, and cancer, where exosome-based therapeutics have shown the most promise. Ultimately, interdisciplinary collaboration across nanotechnology, molecular biology, and clinical medicine will accelerate the transition of exosome-based drug-delivery systems from bench to bedside.

## 9. Conclusions

Exosomes represent a unique subpopulation of natural nanoparticles, functioning as nanocarriers for transferring lipids, proteins, mRNA, non-coding RNA, and DNA, facilitating intercellular communication in various biological processes. In addition to milk-derived exosomes, PDENs might provide a basis for the development of a new class of nanocarriers that could improve drug bioavailability and target delivery. Although the study of exosomes is still in the infancy stage, the similarities in composition and functional characteristics between mammalian-derived exosomes and PDENs suggest that they hold great promise as transport systems for therapy and disease diagnosis. Yet, despite numerous studies on PDENs, our current understanding of the production from different sources, PDENs’ biological mechanisms, and PDENs’ therapeutic potential is still limited, warranting further research.

## Figures and Tables

**Table 1:** Therapeutic applications of exosomes and their mechanisms of action.

Therapeutic Effect	Mechanism of Action	References
Anti-inflammatory	Stable RNA transport via milk exosomes to immune cells	[1]
Targeted drug delivery	Encapsulation of therapeutic agents in exosomes	[13]
Anti-inflammatory	Modulation of inflammatory pathways in immune cells	[17]
Anticancer	Delivery of doxorubicin and targeting colon cancer cells	[37]
Antioxidant, anticancer	Intrinsic bioactive compounds in ginger nanoparticles	[38]
Drug delivery	Utilization of natural plant lipids for systemic distribution	[39]
Enhanced chemotherapy efficacy	Exosomal encapsulation of paclitaxel for sustained release	[24]
Immunomodulation	Expression of TGF-β on vesicle surface	[11]
Neurological targeting	Enhanced targeting of exosomes to brain tissues	[10]
Enhanced bioavailability	Encapsulation of polyphenols for sustained release	[40]

**Table 3:** Overview of clinical research on exosome-based therapies.

Description of Clinical Trial	Possible Mechanisms of Action	Disease Treated	References
MSC-derived exosomes for acute ischemic stroke	Immunomodulation, reduction in inflammation, and neuroprotection via paracrine signaling	Acute ischemic stroke	[100]
Exosome-based delivery of KRAS G12D siRNA (iExosomes)	Targeted gene silencing of KRAS G12D oncogene in pancreatic cancer cells	Pancreatic cancer	[101]
Dendritic-cell-derived exosomes pulsed with tumor antigens	Activation of anti-tumor immune responses via antigen presentation	Non-small-cell lung cancer (NSCLC)	[102]
MSC-derived exosomes for graft-versus-host disease (GvHD)	Immunosuppressive and anti-inflammatory activity through miRNA delivery	Graft-versus-host disease	[103]
Curcumin-loaded exosomes for inflammation control in inflammatory bowel disease (IBD)	Enhanced bioavailability and delivery of anti-inflammatory compounds	Inflammatory bowel disease (IBD)	[104]

## Data Availability

No new data were created or analyzed in this study. Data sharing is not applicable to this article.
